# Function and Regulation of Ferredoxins in the Cyanobacterium, *Synechocystis* PCC6803: Recent Advances

**DOI:** 10.3390/life4040666

**Published:** 2014-11-07

**Authors:** Corinne Cassier-Chauvat, Franck Chauvat

**Affiliations:** UMR8221, CEA, CNRS, Université Paris-Sud, Institut de Biologie et Technologie Saclay, Laboratoire de Biologie et Biotechnologie des Cyanobactéries, CEA-Saclay, Gif sur Yvette 91190, France; E-Mail: franck.chauvat@cea.fr

**Keywords:** cyanobacteria, ferredoxins, iron-sulfur cluster, electron transfer, oxidative stress, metal stress, regulation

## Abstract

Ferredoxins (Fed), occurring in most organisms, are small proteins that use their iron-sulfur cluster to distribute electrons to various metabolic pathways, likely including hydrogen production. Here, we summarize the current knowledge on ferredoxins in cyanobacteria, the prokaryotes regarded as important producers of the oxygenic atmosphere and biomass for the food chain, as well as promising cell factories for biofuel production. Most studies of ferredoxins were performed in the model strain, *Synechocystis* PCC6803, which possesses nine highly-conserved ferredoxins encoded by monocistronic or operonic genes, some of which are localized in conserved genome regions. Fed1, encoded by a light-inducible gene, is a highly abundant protein essential to photosynthesis. Fed2-Fed9, encoded by genes differently regulated by trophic conditions, are low-abundant proteins that play prominent roles in the tolerance to environmental stresses. Concerning the selectivity/redundancy of ferredoxin, we report that Fed1, Fed7 and Fed9 belong to ferredoxin-glutaredoxin-thioredoxin crosstalk pathways operating in the protection against oxidative and metal stresses. Furthermore, Fed7 specifically interacts with a DnaJ-like protein, an interaction that has been conserved in photosynthetic eukaryotes in the form of a composite protein comprising DnaJ- and Fed7-like domains. Fed9 specifically interacts with the Flv3 flavodiiron protein acting in the photoreduction of O_2_ to H_2_O.

## 1. Introduction

Ferredoxins (Fed) are small, mostly acidic, soluble proteins found ubiquitously in biological organisms. They possess a highly negative redox potential and use their iron-sulfur cluster to act as electron distributors in various metabolic pathways. The Fed proteins can be classified according to the nature of their iron-sulfur center ([2Fe-2S], [3Fe-4S] and [4Fe-4S]) and the organisms in which they were isolated for the first time [1]. Hence, the ferredoxins with a [2Fe-2S] cluster can be divided into plant-type or bacterial-type Feds. In plants, algae and cyanobacteria, *i.e.*, prokaryotes with a plant-like oxygen-evolving photosynthetic apparatus, the most abundant ferredoxin, the plant-type [2Fe-2S] Fed, designated as Fed1, is recognized primarily as the protein that mediates electron transfer from iron-sulfur centers of photosystem I (PSI-C subunit) to ferredoxin NADP reductase, which, in turn, reduces NADP^+^ for CO_2_ fixation [2]. Fed1 is also involved in other redox processes, such as cyclic photophosphorylation, nitrogen assimilation, biosynthesis of glutamate and chlorophyll, sulfite reduction, fatty acid metabolism and the modulation of the activity of various enzymes via the thioredoxin system [2]. Furthermore, a Fed1-like domain containing bacteriocin, named “peptocin M2”, was recently shown to parasitize the cell entry and Fed-dependent iron acquisition system of the plant pathogens, *Pectobacterium* spp. [3].

Like other photosynthetic organisms, cyanobacteria possess various [2Fe-2S] and [4Fe-4S] Feds that have received less attention than Fed1, so far [2]. The interest in these ferredoxins has increased by the recent *in vitro* indications that one of these Fed can directly reduce NiFe-hydrogenase, which can produce hydrogen in some conditions [4]. In this review, we summarize what is known about the function and regulation of ferredoxins in cyanobacteria, emphasizing the unicellular model cyanobacterium, *Synechocystis* PCC6803, where the Fed proteins have been mostly studied.

## 2. The Nine Ferredoxins of *Synechocystis* Are Highly Conserved in Cyanobacteria

**Figure 1:**
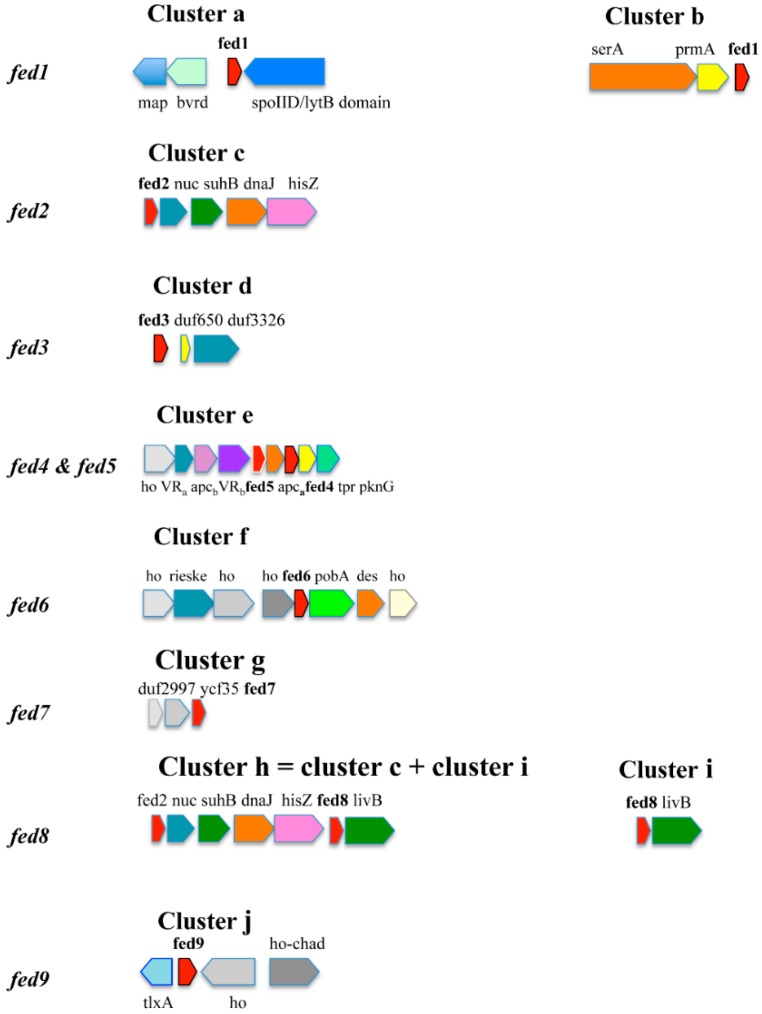
Conserved genomic organization around the ferredoxin genes of the cyanobacterial genomes.

*Synechocystis* (hereafter *Synechocystis*) possesses a small sequenced genome [5], easily manipulable [6,7,8], which encodes nine Feds representative of the ferredoxin diversity. The *fed1-6* genes encode [2Fe-2S] ferredoxins; *fed7* encodes a [4Fe-4S] protein; *fed8* encodes a [3Fe-4S] [4Fe-4S] Fed; and *fed9* encodes a [4Fe-4S] [4Fe-4S] ferredoxin. In agreement with the pivotal role of Feds in electron transfer, all nine *Synechocystis fed*-encoding genes appeared to be highly conserved in cyanobacteria (Table 1). Furthermore a large number of the *fed* genes belong to well-conserved gene clusters (Figure 1), indicating that they operate in specific conserved functions relating to cyanobacterial life.

**Table 1:** Distribution of ferredoxin-encoding genes in cyanobacteria.

	Occurrence of Ferredoxin-Encoding Genes									
	[2Fe-2S]							[3Fe-4S] and [4Fe-4S]		
Cyanobacterial Species	Plant-like					Bacterial-type				
	∑	*fed1*	*fed2*	*fed3*	*fed4*	*fed5*	*fed6*	*fed7*	*fed8*	*fed9*
*Gloeobacter kilaueensis JS1*	3	+	+	−	−	−	−	+ ^g^	+	+
*Gloeobacter violaceus PCC7421*	2	+	+	−	−	−	−	+ ^g^	+	+
*Anabaena cylindrica PCC7122*	5	+ ^a^	+ ^c^	+ ^d^	−	−	+	+ ^g^	+ ^h^	+ ^j^*
*Anabaena* sp. *90*	4	+	+	+ ^d^	−	−	−	+ ^g^	+ ^h'^	+ ^j^*
*Anabaena variabilis ATCC29413*	6	+ ^a^	+ ^c^	+ ^d^	−	−	−	+ ^g^	+ ^h^	+ ^j^
*Cylindrospermum stagnale PCC7417*	6	+ ^a^	+ ^c^	+ ^d^	−	−	+ ^f'^	+ ^g^	+ ^h^	+ ^j^
*Nostoc punctiforme PCC73102*	6	2 ^a^	+	+ ^d^	−	−	2/1 ^f'^	+ ^g^	+ ^h^	+
*Nostoc* sp. *PCC7107*	4	+ ^a^	+	+ ^d^	−	−	−	+ ^g^	+ ^h'^	+ ^j^
*Nostoc* sp. *PCC7120*	4	+ ^a^	+ ^c^	+ ^d^	−	−	+	+ ^g^	+ ^h^	+ ^j^
*Nostoc* sp. *PCC7524*	4	+ ^a^	+ ^c^	+ ^d’^	−	−	+ ^f’^	+ ^g^	+ ^h^	+
*Nostoc azollae 0708*	5	+ ^a^	+ ^c^	+ ^d^	−	−	+	+ ^g^	+ ^h^	+ ^j^*
*Calothrix* sp. *PCC6303*	4	+ ^a’^	+	+	−	−	+	+ ^g^	+ ^h''^	+
*Calothrix* sp. *PCC7507*	6	2 ^a^	+	+ ^d^	−	−	+	+ ^g^	+	+ ^j^
*Rivularia* sp. *PCC7116*	6	2 ^a'^	+ ^c^	+	−	−	+ ^f^	+ ^g^	+	+ ^j''^
*Acaryochloris marina MBIC11017*	8	3	+ ^c^	+	−	5/3 ^e^*	−	+ ^g^	+ ^i^	+
*Chamaesiphon minutus PCC6605*	5	+ ^a'^	+	+	−	+	−	+ ^g^	+	+
*Cyanobacterium aponinum PC 10605*	4	+ ^a'^	+	+	−	+ ^e^*	+ ^f^	+ ^g^	+ ^i^	+
*Cyanobacterium stanieri PCC7202*	4	+	+	+	−	− ^e^*	−	+ ^g^	+ ^i^	+ ^j''^
*Cyanobium gracile PCC6307*	4	+	+	+	−	−	−	+ ^g^	+ ^h^	+ ^j''^
*Cyanothece* sp. *ATCC51142*	7	2 ^a'^	+	+	+ ^e^	+ ^e^	−	+ ^g^	+ ^i^	+ ^j''^
*Cyanothece* sp. *PCC7424*	5	+ ^a'^	+	+	+ ^e^	+ ^e^	−	+ ^g^	+ ^i^	+ ^j''^
*Cyanothece* sp. *PCC7425*	5	+	+ ^c'^	+	−	+ ^e^*	−	+ ^g^	+	+
*Cyanothece* sp. *PCC7822*	7	2 ^a'^	+ ^c'^	+	+ ^e^	+ ^e^	−	+ ^g^	+ ^i^	+
*Cyanothece sp.* *PCC8801*	5	+	+ ^c''^	+	+ ^e^	+ ^e^	−	+ ^g^	+ ^i^	+ ^j''^
*Cyanothece* sp. *PCC8802*	5	+	+ ^c''^	+	+ ^e^	+ ^e^	−	+ ^g^	+ ^i^	+ ^j''^
*Dactylococcopsis salina PCC8305*	3	+	+	+	−	−	−	+ ^g^	+ ^i^	+
*Gloeocapsa* sp. *PCC7428*	7	+ ^a'^	+	+	−	+ ^e^*	−	+ ^g^	+	+ ^j''^
*Halothece* sp. *PCC7418*	4	+	+	+	−	−	−	+ ^g^	+ ^i^	+ ^j''^
*Microcystis aeruginosa NIES-843*	4	2	+ ^c''^	+	−	−	−	+ ^g^	+	+ ^j''^
*Synechococcus elongatus PCC6301*	3	+ ^b^	+	+	−	−	−	+ ^g^	+	+ ^j''^
*Synechococcus elongatus PCC7942*	3	+ ^b^	+	+ ^d^	−	−	−	+ ^g^	+	+ ^j''^
*Synechococcus* sp. *CC9311*	6	2 ^b^	+	+ ^d^	−	−	−	+ ^g^	+	−
*Synechococcus* sp. *CC9605*	6	+ ^b^	+	+ ^d^	−	−	−	2 ^g^	+	−
*Synechococcus* sp. *CC9902*	5	+ ^b^	+	+ ^d^	−	−	−	+ ^g^	+	−
*Synechococcus JA-2-3B' a(2-13)*	4	+	+	+	−	2	−	−	+	+
*Synechococcus JA-3-3B' Ab*	4	+	+	+	−	2	−	−	+	+
*Synechococcus* sp. *PCC 6312*	4	+	+	+ ^d^	−	+ ^e*^	−	+ ^g^	+	+
*Synechococcus* sp. *PCC7002*	4	2 ^a'^	+	+	−	2	−	+ ^g^	+	+
*Synechococcus* sp. *PCC7502*	4	+	+	+	−	3	−	+ ^g^	+	+
*Synechococcus* sp. *RCC307*	4	+ ^b^	+ ^c''^	+ ^d^	−	−	−	+ ^g^	+	+ ^j''^
*Synechococcus* sp. *WH7803*	4	+ ^b^	+ ^c'''^	+ ^d^	−	−	−	+ ^g^	+	−
*Synechococcus* sp. *WH8102*	4	+ ^b^	+^c'''^	+ ^d^	−	−	−	+ ^g^	+	−
*Synechocystis* sp. *PCC6803*	4	+	+ ^c''^	+	+ ^e'^	+ ^e'^	+ ^f*^	+ ^g^	+ ^i^	+
*Thermosynechococcus elongatus BP1*	4	+	+	+	−	+ ^e''^	−	+ ^g^	+	+
*Cyanobacterium UCYN-A*	4	+	+	+	−	−	−	−	+	−
*Arthrospira platensis NIES-39*	3	+	+ ^c^	+ ^d^	−	+	−	+ ^g^	+	+ ^j''^
*Crinalium epipsammum PCC9333*	4	+ ^a''^	+ ^c^	+ ^d^	−	−	+ ^f^	+ ^g^	+	+ ^j''^
*Geitlerinema* sp. *PCC7407*	3	+ ^a''^	+ ^c^	+ ^d^	−	+	+ ^f^	+ ^g^	+	+
*Leptolyngbya* sp. *PCC7376*	4	2 ^a''^	+ ^c^	+	−	+	−	−	+	+
*Microcoleus* sp. *PCC7113*	6	2	+ ^c^	+	−	−	+^f^	+ ^g^	+	+ ^j''^
*Oscillatoria acuminata PCC6304*	3	+ ^a’^	+ ^c^	+ ^d^	−	+	−	+ ^g^	+	+ ^j''^
*Oscillatoria nigroviridis PCC7112*	4	+ ^a’^	+ ^c^	+	−	−	+^f^	+ ^g^	+	+ ^j''^
*Pseudanabaena* sp. *PCC7367*	4	+	+	+	−	+	−	+ ^g^	+	+
*Trichodesmium erythraeum ISM101*	4	3 ^a''^	−	+	−	−	−	+ ^g^	+	+ ^j''^
*Chroococcidiopsis thermalis PCC7203*	5	+	+	+ ^d^	−	+ ^e''^	−	+ ^g^	2	+ ^j''^
*Pleurocapsa* sp. *PCC7327*	5	+	+ ^c'^	+	+ ^e'^	+ ^e^	−	+ ^g^	+ ^i^	+ ^j''^
*Stanieria cyanosphaera PCC7437*	4	+	+	+	+ ^e'^	+ ^e'^	−	+ ^g^	+ ^i^	+ ^j''^
*Prochlorococcus marinus AS9601*	3	+ ^b^	+ ^c'''^	+ ^d^	−	−	−	+ ^g'^	−	−
*Prochlorococcus marinus MIT9211*	2	+ ^b^	+ ^c'''^	−	−	−	−	+ ^g'^	+	−
*Prochlorococcus marinus MIT9215*	2	+ ^b^	+ ^c'''^	?	−	−	−	+ ^g'^	−	−
*Prochlorococcus marinus MIT9301*	3	+ ^b^	+ ^c'''^	+ ^d^	−	−	−	+ ^g'^	−	−
*Prochlorococcus marinus MIT9303*	1	+ ^b^	−	−	−	−	−	+ ^g'^	−	−
*Prochlorococcus marinus MIT9312*	3	+ ^b^	+ ^c'''^	+ ^d^	−	−	−	+ ^g'^	−	−
*Prochlorococcus marinus MIT9313*	1	+ ^b^	−	−	−	−	−	+ ^g'^	−	−
*Prochlorococcus marinus MIT9515*	2	+ ^b^	+	−	−	−	−	+ ^g'^	−	−
*Prochlorococcus marinus NATL1A*	3	+ ^b^	+	−	−	−	−	+ ^g'^	−	−
*Prochlorococcus marinus NATL2A*	3	+ ^b^	+	−	−	−	−	+ ^g'^	−	−
*Prochlorococcus marinus SS120*	2	+ ^b^	+	−	−	−	−	+ ^g'^	+	−
*Prochlorococcus marinus MED4*	3	+ ^b^	+ ^c’’^	+ ^d^	−	−	−	+ ^g'^	−	−

The nine *fed* genes are designated as follows in *Synechocystis*: *fed1* (ssl0020) [5], *fed2* (sll1382), *fed3* (slr1828), *fed4* (slr0150), *fed5* (slr0148), *fed6* (ssl2559), *fed7* (sll0662), *fed8* (ssr3184) and *fed9* (slr2059). The presence or absence in cyanobacteria of the *Synechocystis* orthologous *fed* genes is indicated by +, along the indicated numbers, or −, respectively. The superscript letters indicate those *fed* genes located in well-conserved genome organization contexts, which are depicted in Figure 1. **∑** indicates the total number of plant-like ferredoxin genes.

The genes are represented by boxes pointing in the direction of their transcription. Hypothetical genes are indicated as “ho”. Cluster **a** = methionine aminopeptidase (map); putative biliverdin reductase (bvdR); fed1; spoIID/lytB domain-containing protein. Cluster **a'** = Cluster **a** with no map gene. Cluster **b** = D-3-phosphoglycerate dehydrogenase (serA), ribosomal protein L11 methyltransferase (prmA), fed1. Cluster **c** = fed2; hypothetical nuclease (nuc); inositol-1-monophosphatase (suhB); heat shock protein DnaJ domain-containing protein (dnaJ); ATP phosphoribosyl-transferase regulatory subunit (hisZ). Cluster **c'** = Cluster **c** with no nuc gene. Cluster **d** = fed3; conserved hypothetical protein DUF650 (duf650); conserved hypothetical protein DUF3326 (duf3326). Cluster **e** = hypothetical (ho); 4-vinyl reductase-like protein (4VR_a_); protein harboring homology with allophycocyanin beta subunit (apc_b_); 4-vinyl reductase (4VR_b_); fed4; protein similar to allophycocyanin alpha subunit (apc_a_); fed5; TPR domain containing protein (TPR). Cluster **e'** = Cluster **e** + threonine protein kinase (pknG). Cluster **e''** = Cluster **e'** without fed4.

Cluster e* = Cluster **e** lacking fed4. Cluster **f** = hypothetical protein (ho); ring-hydroxylating dioxygenase (2Fe-2S) large terminal subunit (rieske); hypothetical protein (ho); hypothetical protein (ho); fed6; ring-hydroxylating dioxygenase (2Fe-2S) domain-containing protein phenoxybenzoate dioxygenase subunit (pobA); sterol desaturase (des); hypothetical protein (ho). Cluster **f'** = Cluster **f** with two hypothetical protein-encoding genes (ho) located between fed6 and pobA. Cluster **g** = DUF2997 domain-containing hypothetical protein (duf2997); DUF1257 domain-containing hypothetical protein (ycf 35); fed7. Cluster **h** = Cluster **c** + Cluster **i**. Cluster **h'** = Cluster **c** lacking fed2; nuc and suhB + Cluster **i**. Cluster **h''** = Cluster **c** missing fed2 and nuc + Cluster **i**. Cluster **i** = fed8; ABC-type branched-chain amino acid transport systems; ATPase component (livB). Cluster **j** = thiol-disulfide interchange protein (TxlA); Fed9; hypothetical protein (ho); CHAD domain-containing protein (chad). Cluster j* = **j** missing ho. Cluster **j'** = Cluster **j** without chad. Cluster **j''** = Cluster **j** lacking both ho and chad.

## 3. The *Synechocystis* Ferredoxins Genes Are Differently Regulated by Trophic Conditions

The influence of environmental conditions on the expression of the ferredoxin genes was analyzed by northern blot and/or DNA microarrays experiments, as well as transcriptional fusion to a reporter gene in the case of the *fed1* gene (ssl0020, also named *petF*). The *fed1* monocistronic transcripts, which are abundant in standard photoautotrophic conditions [9], in agreement with the high content of the Fed1 protein [10], become scarce after a 15-min shift to darkness. They reappeared rapidly (in 5 min) and strongly after re-illumination in a process requiring *de novo* transcription and active photosynthesis, similarly to what occurs in plants [11]. In *Synechocystis*, this regulation occurs, at least in part, at the level of the complex promoter, which possesses several light-responsive elements promoting strong light induction [11]. The expression of the *fedI* gene is also positively regulated by carbon (NaHCO_3_) availability [11], under the control of NdhR [11], a key regulator of carbon assimilation [12]. Furthermore, *fed1* is negatively regulated by hydrogen peroxide (H_2_O_2_), cadmium, iron starvation the photosynthetic inhibitors, DCMU (3-(3,4-dichlorophenyl)-1,1-dimethylurea) and DBMIB (2,5-dibromo-3-methyl-6-isopropyl-*p*-benzoquinone), selenate, selenite and zinc (Table 2).

**Table 2:** Regulation of the *Synechocystis* ferredoxin genes in response to environmental challenges.

Name	Conditions Triggering Upregulation of the *Fed* Genes	Conditions Triggering Downregulation of the *Fed* Genes
*fed1*	Light [9,11]; NaHCO_3_ [11];	Darkness [9]; Cd, LFe, H_2_O_2_ [11,13]; Na_2_SeO_3_, Na_2_SeO_4_ [11]; HZn [13]; DCMU, DBMIB, LiC, HT°, SS [14]
*fed2*	Cd, H_2_O_2_, HZn [13]; HL, BL, UV, SS [14]	Glc [9]; Na_2_SeO_4_ [11]; LiC [14]
*fed3*	BL* [14]	Cd [13]; H_2_O_2_ [13,14]; LiC [14]
*fed4*	LL [9]; H_2_O_2_ [13]	Cd, LFe, HZn [13]; HL, DCMU, LiC, SS [14]
*fed5*	LL [9]; H_2_O_2_ [13]	Cd, LFe, HZn [13]; HL, DCMU, LiC, SS [14]
*fed6*	BL [14]	
*fed7*	LFe [13]; HL [14] LiC [15]	H_2_O_2_ [11,13]; Cd, HFe [13]
*fed8*	Cd [11]; HL, LiC [14]	H_2_O_2_, LFe [14]
*fed9*	HL, HT° [14]	

Note: BL, blue light; BL*, blue light (one out of the six time points); Cd, cadmium; DCMU, (3-(3,4-dichlorophenyl)-1,1-dimethylurea); DBMIB, 2,5-dibromo-3-methyl-6-isopropyl-*p*-benzoquinone; Glc, glucose; H_2_O_2_, hydrogen peroxide; LiC, inorganic carbon limitation; HFe, high iron; LFe, iron starvation; HL, high light; LL, low light; SS, salt stress; HT°, high temperature; HZn, high zinc. *fed4* and *fed5* belong to the slr0144-slr0151 octacistronic operon, while *fed7* belongs to the ssl0060-ssl1263-ssl0662 tricistronic operon.

In contrast to *fed1*, the other *fed* genes of *Synechocystis* are weakly expressed under standard photoautotrophic conditions [9,11,13]. These observations are consistent with the fact that their products were undetected in *Synechocystis* protein extracts, unlike Fed1 [10]. Similarly to *fed1*, the eight other *fed2-9* genes are regulated by environmental conditions (Table 2).

## 4. The Nine *Synechocystis* Ferredoxins Play a Crucial Role in Photoautotrophic Growth or Tolerance to Environmental Stresses

To investigate the nine fed genes, we independently replaced each fed coding sequence (fed-CS) with a transcription terminator-less marker, Km^r^, for antibiotic selection, while maintaining their DNA flanking regions (about 300 bp) to serve for homologous recombinations mediating targeted gene replacement upon transformation to *Synechocystis* [16]. The resulting deletion cassettes (Δ*fed1*::Km^r^, Δ*fed2*::Km^r^, Δ*fed3*::Km^r^, Δ*fed4*::Km^r^, Δ*fed5*::Km^r^, Δ*fed2*::Km^r^, Δ*fed3*::Km^r^, Δ*fed4*::Km^r^ and Δ*fed9*::Km^r^) were independently introduced by transformation in *Synechocystis*, which harbors about 10 chromosome copies per cell [16]. We verified through PCR and DNA-sequencing that the marker gene had been properly inserted in the *Synechocystis* chromosome, in place of the studied gene, and we assayed whether the segregation between wild-type (WT) and mutant (Δ*fed*) chromosome copies was complete (the studied *fed* gene is dispensable to cell growth) or not (the studied gene is essential to cell viability).

The Δ*fed1*::Km^r^/*fed1*^+^ mutant growing under photoautotrophic condition harbored both WT (*fed1*^+^) and mutant (Δ*fed1*::Km^r^) chromosomes, irrespective of the duration (≥100 generations) and dose (up to 300 µg·mL^−1^) of the Km^r^ selection. This result showed that *fed1* is essential to the photoautotrophic growth of *Synechocystis* [9], as observed in the obligate photoautotroph cyanobacterium, *Synechococcus* PCC7942 [17]. The *Synechocystis fed1* gene was found to be also crucial in cells growing in the presence of glucose, which supports cell growth in the absence of photosynthesis [9].

Like *fed1*, the *fed2*, *fed3*, *fed6* and *fed8* genes appeared to be essential to the photoautotrophic growth of *Synechocystis* (Table 3).

**Table 3:** Characteristics of the ferredoxin-encoding genes in *Synechocystis*.

Name	Gene ID	Type of Iron Sulfur Center	Importance for Photo-Autotrophic Growth	Reference
*fed1*	ssl0020	[2Fe-2S] plant-like	Essential	[4,9]
*fed2*	sll1382	[2Fe-2S] plant-like	Essential	This study
*fed3*	slr1828	[2Fe-2S] plant-like	Essential	This study
*fed4*	slr0150	[2Fe-2S] plant-like	Dispensable	This study, [4]
*fed5*	slr0148	[2Fe-2S] adrenodoxin-like	Dispensable	This study
*fed6*	ssl2559	[2Fe-2S] bacterial type	Essential	This study
*fed7*	sll0662	[4Fe-4S]	Dispensable	[15,18]
*fed8*	ssr3184	[3Fe-4S] [4Fe-4S]	Essential	This study
*fed9*	slr2059	[4Fe-4S] [4Fe-4S]	Dispensable	This study

**Figure 2:**
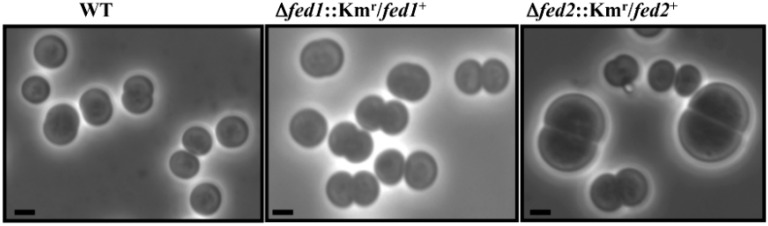
Typical morphology of *Synechocystis* wild-type (WT) and mutant cells depleted in the essential plant-like ferredoxins, Fed1 (Δ*fed1*::Km^r^/*fed1*^+^) or Fed2 (Δ*fed2*::Km^r^/*fed2*^+^).

Note: Scale bar = 1 µm.

The cells depleted of either Fed3 or Fed8 were killed by a prolonged exposure to Km and could not be further studied, whereas the Fed2-depleted cells (Δ*fed2*::Km^r^/*fed2*^+^) appeared to display an increased size as compared to WT or Fed1-depleted cells (Figure 2).

The four *fed* mutants, Δ*fed4*::Km^r^, Δ*fed5*::Km^r^, Δ*fed7*::Km^r^ and Δ*fed9*::Km^r^, retained no WT chromosome copies and grew healthily in photoautotrophic conditions. These findings indicate that the *fed4*, *fed5*, *fed7* and *fed9* genes are dispensable for *Synechocystis* growth, in agreement with previous reports on *fed4* [4] and *fed7* [15,18]. The complete absence of WT chromosomes in each mutant was also verified in cultures subsequently grown for about 100 generations in the absence of the Km antibiotic to stop counter-selecting the propagation of possibly remaining wild-type (WT) chromosome copies, prior to the PCR assays. The absence of WT chromosome copies confirmed that the *fed4*, *fed5*, *fed7* and *fed9* genes are dispensable for the viability of *Synechocystis* (Table 3).

### Fed7 and Fed9 Ferredoxins Plays a Prominent Role in the Tolerance to Oxidative and Metal Stresses, and the [2Fe-2S] Center of Fed7 Is Required for the Tolerance to Iron Starvation

**Figure 3:**
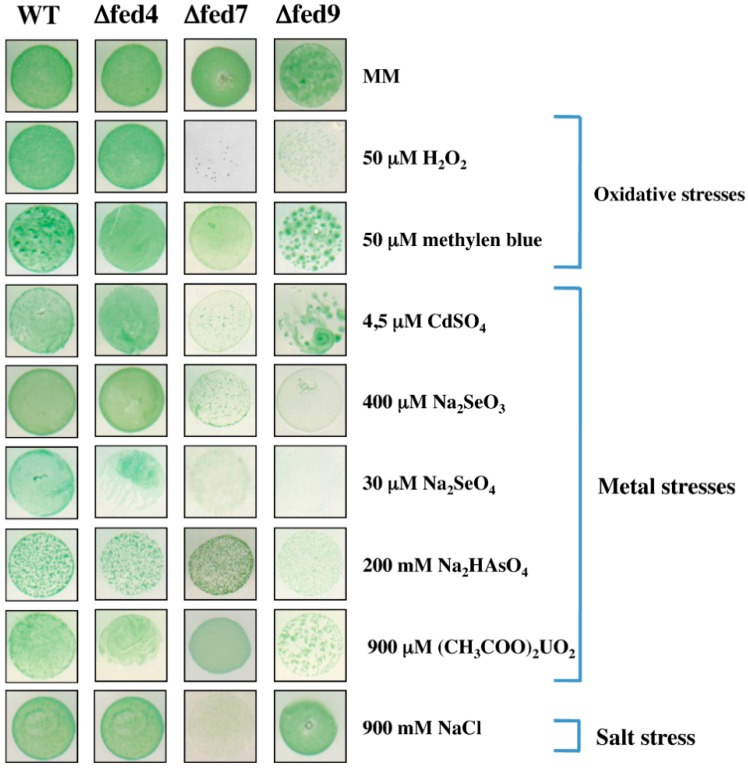
Influence of the dispensable ferredoxins on the tolerance of *Synechocystis* to various environmental stresses.

As the mutants with *fed4*, *fed5*, *fed7* or *fed9* deleted grow well in standard photoautotrophic conditions, it was possible to investigate their tolerance to environmental stresses. In search of ferredoxin selectivity, we found that the absence of Fed7 or Fed9, but neither Fed4 nor Fed5, decreases the tolerance to oxidative and metal stresses (Figure 3). Furthermore, only Fed7 appeared to be involved in the protection against salt stress (Figure 3).

Ten-microliter aliquots of exponentially growing cells (2.5 × 10^7^ cells·mL^−1^) of the strains WT (wild-type), Δ*fed4* (*fed4* null mutant), Δ*fed7* (*fed7* null mutant) and Δ*fed9* (*fed9* null mutant) were spotted onto solid mineral medium (MM) with or without the indicated concentration of the tested agents. The plates were subsequently incubated for 4–5 days in standard photoautotrophic conditions at 30 °C, prior to image acquisition. As it is indistinguishable from that of Δ*fed4*, the phenotype of the Δ*fed5* mutant is not shown. These experiments were performed at least three times.

**Figure 4:**
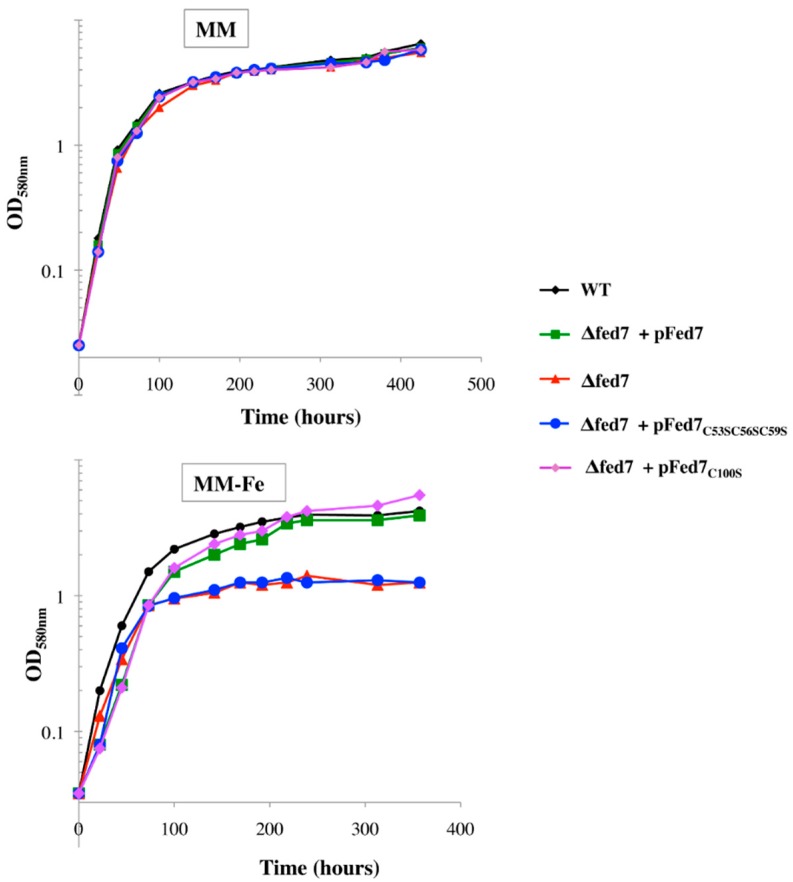
Influence of Fed7 and its [2Fe-2S] center on the tolerance of *Synechocystis* to iron limitation.

Because *fed7* appeared to be induced by iron starvation (Table 2), we anticipated that Fed7 is required for protection against iron limitation. Indeed, the Δ*fed7*::Km^r^ null mutant appeared to be susceptible to iron limitation, and this phenotype could be rescued by plasmid complementation, as follows. The *fed7* protein coding sequence (Table 1) was cloned into the pFC1 plasmid, which replicates autonomously in *Synechocystis* at the same copy number as the polyploid chromosome and expresses the studied genes proportionally to the growth temperature [8]. As expected, the moderate production of Fed7 (driven by the pFed7 plasmid) in cells incubated at 34 °C rescued the otherwise low tolerance to iron limitation of the Δ*fed7*::Km^r^ mutant back to the WT level (Figure 4). Using the same strategy, we found that the cysteine to serine substitution at position 100 in the Fed7 amino-acid sequence did not impair the rescue complementation (compare the strains Δ*fed7* with or without the plasmids pFed7 or pFed7_C100S_). By contrast, the triple mutation of cysteines 53, 56 and 59, which coordinates the [2Fe2S] center of Fed7 (together with C96), abolished the complementation (compare the strains Δ*fed7* with or without the plasmids pFed7 or pFed7_C53S,C56S,C59S_), showing that the [2Fe2S] center of Fed7 is required for the tolerance to iron starvation. These data show that Fed7 operates in tolerance of iron limitation, likely by constituting a redox-responsive element.

Typical growth of WT cells (black symbols) and mutant Δfed7 (red triangles), Δfed7 + pFed7 (green squares), Δfed7 + pFed7_C53S56SC59S_ (blue circles) and Δfed7 + pFed7_C100S_ (purple diamonds) cultivated for the indicated durations in standard liquid mineral medium (MM, which contains 17 µM Fe provided as ferric ammonium citrate) or in iron-limited medium (MM lacking ferric ammonium citrate, which contains only trace amounts of Fe) are shown. All experiments were performed at least three times at 34 °C to allow moderate expression of the various *fed7* alleles from the replicating plasmid pFC1 of the f*ed7* (see above).

## 5. Analysis of the Selectivity/Redundancy of Ferredoxins: Identification of Fed-Interacting Proteins

To identify proteins that can physically interact with one or several Feds, we used the bacterial adenylate cyclase two-hybrid (BACTH) system [19], exactly as we described [18,20,21]. The full-length coding sequences of the Feds and possible redox partners were translationally fused to the intrinsically-inactive adenylate cyclase domains of the replication-compatible BACTH reporter plasmids, pKT25 and pUT18. The resulting pUT18 and pKT25 derivatives were doubly-transformed to the *E. coli* reporter strain, DHM1, to search for protein-protein interaction that reconstituted adenylate cyclase, which turned on β-galactosidase. Several of these Fed-partner interactions were verified through the identification of interaction-disruptive mutations in each protein partner (Table 4).

**Table 4:** Identification and analysis of Fed-interacting proteins with the bacterial adenylate cyclase two-hybrid (BACTH) system.

Gene Cloned in pUT18	Gene Cloned in pKT25	β-GAL Activity (nmol·min^−1^·mg^−1^)	Reference
Controls:			
zip domain	zip domain	4213 ± 385	This study
none	none	75 ± 4	This study
fed7	none	74 ± 8	This study
none	ftrC	82 ± 4	[18]
fed9	none	76 ± 7	This study
none	fed9	69 ± 6	This study
Tests:			
fed7	fed9	92 ± 8	This study
fed7	dnaJ	1081 ± 88	This study
fed7_C53S_	dnaJ	346 ± 20	This study
fed7_C53S C56S C59S_	dnaJ	413 ± 69	This study
fed7_C100S_	dnaJ	781 ± 26	This study
fed9	dnaJ	78 ± 6	This study
fed7	ftrC	1766 ± 164	[18]
fed7_C53S_	ftrC	567 ± 87	This study
fed7_C53S C56S C59S_	ftrC	587 ± 104	This study
fed7_C96S_	ftrC	652 ± 47	This study
fed7_C100S_	ftrC	237 ± 32	This study
fed7	ftrC_C31S_	1428 ± 16	This study
fed7	ftrC_C56S_	1460 ± 52	This study
fed7	ftrC_C58S_	228 ± 32	[18]
fed7	ftrC_C75S_	1597 ± 116	This study
fed7	ftrC_C77S_	1223 ± 17	This study
fed7	ftrC_C86S_	1475 ± 24	This study
fed7	ftrC_C88S_	1613 ± 231	[18]
fed9	ftrC	2728 ± 184	This study
fed9_C84S C87S C90S C125S_	ftrC	2649 ± 42	This study
fed9_C94S C115S C118S C121S_	ftrC	2058 ± 12	This study
fed9_D80A_	ftrC	73 ± 3	This study
fed9	ftrC_C31S_	2531 ± 128	This study
fed9	ftrC_C56S_	2312 ± 99	This study
fed9	ftrC_C58S_	3181 ± 113	This study
fed9	ftrC_C75S_	2159 ± 53	This study
fed9	ftrC_C77S_	2350 ± 52	This study
fed9	ftrC_C86S_	2241 ± 120	This study
fed9	ftrC_C88S_	121 ± 2	This study
fed7	flv3	89 ± 9	This study
fed9	flv3	2797 ± 175	This study
fed9_C84S C87S C90S C125S_	flv3	2008 ± 123	This study
fed9_C94S C115S C118S C121S_	flv3	2311 ± 28	This study
fed9_D80A_	flv3	2253 ± 84	This study
fed9	fed9	2472 ± 190	This study
fed9	fed9_D80A_	3057 ± 250	This study
fed9	sll0330	1934 ± 42	This study

The occurrence of physical interactions between the Feds and their partner proteins produced from the replication compatible pUT18 and pKT25 BACTH reporter plasmids co-transformed to *E. coli* was ascertained by measuring the β-galactosidase activity (1 β-Gal unit corresponds to the hydrolysis of 1 nmol of *O*-nitrophenyl-β-d-galactopyranoside; min^−1^·mg^−1^ of protein). The numbers are the mean value ± standard deviations of six assays (three measurements performed on two different cell extracts). The plasmids with or without the zip insert served as positive and negative controls, respectively [19]. The nature and position of amino-acid substitutions are written in subscripts. The presumed redox-active cysteines are indicated with superscripted asterisks. The name of the Feds proteins partners are as follows: DnaJ-domain-containing protein, sll1384; Flv3 (flavodiiron protein 3), sll0550; FTRc (ferredoxin-thioredoxin reductase catalytic chain), Sll0554.

### 5.1. Fed1, Fed7 and Fed9 Belong to a Ferredoxin-Glutaredoxin-Thioredoxin Crosstalk Pathway Operating in Stress Resistance

Using a combination of methods (two-hybrid, GST pull-down, western blotting, enzymatic assays and gene deletion and plasmid-rescue complementation in *Synechocystis*), we showed that Fed7 belongs to a complex redox pathway [18]. This pathway sequentially transfers the photosynthetic electrons to Fed1, FTRc (the ferredoxin-thioredoxin reductase catalytic chain), Fed7 and glutaredoxin 2. In addition, glutaredoxin 2 can also receive electrons from the NAD(P)H-thioredoxin reductase-glutaredoxin 1 pathway. The resulting crosstalk pathway plays a crucial role in the protection against hydrogen peroxide and selenate [18].

**Figure 5:**
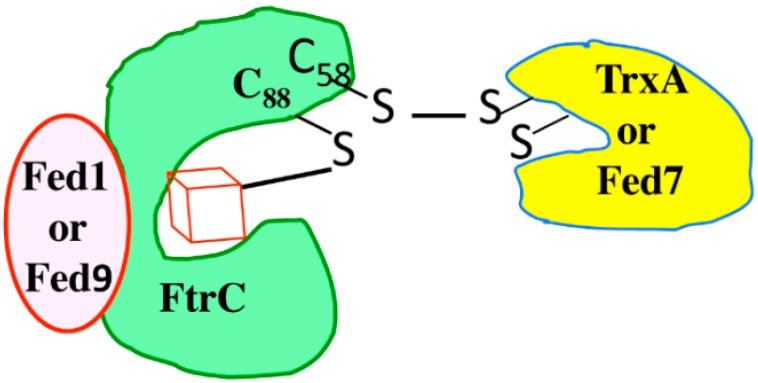
Scheme representating the possible Fed1/Fed9-FtrC-TrxA/Fed7 interactions.

Similarly to Fed1 and Fed7, Fed9 appeared to interact with FTRc (Table 4). From this Fed9-FTRc interaction and the following lines of evidence, we propose that Fed1 and Fed9 interact with the same face of FTRc to reduce it, whereas both Fed7 and thioredoxin (Trx) interact with the other face of FTRc to be reduced by it (Figure 5). First, knowing that electrons are transferred from the most to the least electronegative proteins, it is worth noting that the approximate redox potentials are −420 mV for both Fed1 and Fed9; −350 mV for FTRc; −400 mV or −150 mV for the [4Fe-4S] or the [3Fe-4S] forms of Fed7; and −270 mV for Trx (thioredoxin). Second, it has been shown that Fed1 and Trx bind on opposite sites of the disc-shaped FTRc protein, to form the Fed1-FTRc-TrxA pathway, which transfers electrons in that order [2,22]. Third, the Fed9-FTRc interaction was abolished by the D80A mutation in Fed9 and by the C88S mutation in FTRc, which did not impair the FTRc-Fed7 interaction (Table 4). Fourth, the FtrC-Fed7 interaction was abolished by the C100S mutation in Fed7 and by the C58S mutation in FTRc, which did not alter the Fed9-FTRc interaction (Table 4). By analogy with the Fed1-FTRc-TrxA redox pathway [22], we propose that in the case of oxidative stress, the [4Fe4S] cluster of Fed7 is converted into a [3Fe4S] center, thereby liberating the C56 cysteine that normally operates in the coordination of the [4Fe4S] cluster. The liberated C56 cysteine forms a disulfide bridge with the C100 cysteine, thereby turning the [3Fe4S] form of Fed7 into a TrxA-like protein (Figure 5).

The FtrC protein and its [4Fe-4S] center are represented by the green form and the red cube, respectively, while Fed1 (and Fed9) are represented by the red circle and TrxA (and Fed7) by the yellow form.

### 5.2. Identification of Proteins Selectively Interacting with Either Fed7 or Fed9, but Not Both

As a step towards the identification of selective functions of Fed7 or Fed9, we noticed that Fed7, but not Fed9, physically interacts with Sll1384, a DnaJ-like protein (Table 4), which is dispensable to cell life [23], like Fed7 [15,18]. Our finding is consistent with the occurrence in photosynthetic eukaryotes, such as *Chlamydomonas reinhardtii*, of a DnaJ-Fed composite protein comprising a DnaJ domain (similar to Sll1384) fused to a Fed domain (similar to Fed7, the closest homolog of this Fed domain in *Synechocystis*). Together, these data support the proposal that eukaryotic DnaJ-Fed composite proteins evolved from independent, but physically-interacting DnaJ-like and Fed7-like cyanobacterial proteins [15,24].

Also, interestingly, we found that Fed9, but not Fed7, physically interacts with Sll0550, a non-essential flavodiiron protein (Flv3), which operates in the NAD(P)H-driven photoreduction of O_2_ to H_2_O [25].

## 6. Conclusions

It is important to analyze the selectivity/redundancy of ferredoxins in cyanobacteria, because these enzymes play crucial roles in the growth and/or tolerance to environmental stresses of these fascinating organisms, which produce a large part of the oxygen and biomass for the food chain and also have high biotechnological interest. So far, most of what we know concerning cyanobacterial ferredoxins came from the analysis of the nine ferredoxins of the model strain, *Synechocystis* PCC6803, which are highly conserved in cyanobacteria. However, it is important to prolong and extend those studies by analyzing the ferredoxins of other cyanobacteria that colonize different biotopes (marine waters, desert soils) or perform processes not accomplished by *Synechocystis* (nitrogen fixation, multicellularity and differentiation of specialized cells, such as akinetes and/or heterocysts).
